# Impact of dropout of female volunteer community health workers: An exploration in Dhaka urban slums

**DOI:** 10.1186/1472-6963-12-260

**Published:** 2012-08-17

**Authors:** Khurshid Alam, Jahangir AM Khan, Damian G Walker

**Affiliations:** 1 Centre for Equity and Health Systems, ICDDR,B, 68 Shaheed Tajuddin Ahmed Sharani, Mohakhali, Dhaka, 1212, Bangladesh; 2 Monash School of Public Health & Preventive Medicine, Monash University, 99 Commercial Road, The Alfred Centre, Melbourne, Vic, 3004, Australia; 3 Financial and Health Policy, Global Health Program, Bill and Melinda Gates Foundation, Seattle, USA

**Keywords:** BRAC CHWs, Impact of dropout, Ingredient approach, Friction cost approach, Sustainability, Urban slums

## Abstract

**Background:**

The model of volunteer community health workers (CHWs) is a common approach to serving the poor communities in developing countries. BRAC, a large NGO in Bangladesh, is a pioneer in this area, has been using female CHWs as core workers in its community-based health programs since 1977. After 25 years of implementing of the CHW model in rural areas, BRAC has begun using female CHWs in urban slums through a community-based maternal health intervention. However, BRAC experiences high dropout rates among CHWs suggesting a need to better understand the impact of their dropout which would help to reduce dropout and increase program sustainability. The main objective of the study was to estimate impact of dropout of volunteer CHWs from both BRAC and community perspectives. Also, we estimated cost of possible strategies to reduce dropout and compared whether these costs were more or less than the costs borne by BRAC and the community.

**Methods:**

We used the ‘ingredient approach’ to estimate the cost of recruiting and training of CHWs and the so-called ‘friction cost approach’ to estimate the cost of replacement of CHWs after adapting. Finally, we estimated forgone services in the community due to CHW dropout applying the concept of the friction period.

**Results:**

In 2009, average cost per regular CHW was US$ 59.28 which was US$ 60.04 for an ad-hoc CHW if a CHW participated a three-week basic training, a one-day refresher training, one incentive day and worked for a month in the community after recruitment. One month absence of a CHW with standard performance in the community meant substantial forgone health services like health education, antenatal visits, deliveries, referrals of complicated cases, and distribution of drugs and health commodities. However, with an additional investment of US$ 121 yearly per CHW BRAC could save another US$ 60 invested an ad-hoc CHW plus forgone services in the community.

**Conclusion:**

Although CHWs work as volunteers in Dhaka urban slums impact of their dropout is immense both in financial term and forgone services. High cost of dropout makes the program less sustainable. However, simple and financially competitive strategies can improve the sustainability of the program.

## Background

The emerging consensus is that one of the key ingredients to achieve improved health outcomes is an adequate health workforce [1]. Global estimates suggest a shortage of at least four million health workers [2,3]. Like many other developing countries in the world, there is a scarcity of health human resources in Bangladesh resulting poor provider-population ratio such as 146 health care providers per 10,000 population [4]. This crisis of health human resources is considered as an obstacle to achieving health-related Millennium Development Goals by 2015 [5]. Eventually, such a scarcity of health human resources in Bangladesh is likely contributing to a weaker health system and poor health outcomes.

BRAC, a large NGO in Bangladesh, finds that community members do not have access to affordable high quality medical services leaving them with little or no options for seeking health services [6]. So, BRAC has engaged a large number of volunteer community health workers (CHWs) popularly known as *Shasthya Shebikas*. These CHWs are the core of BRAC’s community-based health interventions, going from door to door and serving as the first point of contact between community members and BRAC health services. The CHWs are selected from their own communities and include both BRAC village organization (VO) members and community members. Currently, about 80,000 CHWs work throughout the country, in both rural and urban areas. Originally developed in rural communities in 1977, in urban areas CHWs participated in the tuberculosis program and later on in the *Manoshi* project which aimed to provide mother, newborn and child health services in urban slums of Bangladesh. Each CHW was responsible for overseeing an average of 200 households and visiting 8–10 households a day. They visited homes to disseminate health messages, identified pregnancies, brought pregnant women to birthing huts, accompanied them during their delivery and provided newborn care.

Globally, the reported attrition rates of CHWs are between 3.2% and 77%, and the high rates are associated with generally with volunteer CHWs [7]. Since the inception of the volunteer CHW model, BRAC has been also struggling with high dropout rates ranging from 20% to 32% depending on the location and the program [8]. The *Manoshi* program had been in operation for about four years in urban slums of Dhaka city where the median duration of employment for the current CHWs was 13 months whereas it was six months for the dropout CHWs. More than one-fifth of the dropout CHWs dropped out in their first month of employment with *Manoshi*; 50% dropped out within six months; 76% within nine months; and more than 90% within the first year [9]. High attrition rates, such as these, have been found to contribute to a decreased stability of programs and increased training costs because of the continuous need for replacement [10]. They have also been shown to have important financial implications for programs. For example, in BRAC’s Essential Health Care program it was estimated that the cost of a dropout CHW was US$ 24 and the opportunity cost was US$ 15 if she had participated in a three-week basic training course, worked for a month, and attended a one-day refresher training [8]. So, in order to better understand dropout of CHWs and its consequences the *Manoshi* program extended its interest in studying retention of CHWs, and impact of dropout of CHWs. Retention was explored in elsewhere [11] which identified some potential strategies to reduce dropout of CHWs. The most commonly discussed recommendation was to address their expectation of income by increasing existing financial incentives e.g., an increased allowance for attending refresher training, an incentive package for pregnancy identification, and supply of drugs and commodities at lower cost. But alternatives like the supply of saris or shoes and free treatment for their family members in sickness were also suggested. Still others suggested bonuses or tips before major festivals and an additional suggestion was the provision of identity cards to make them more recognizable to the community. Secondly, in order to minimize the frustrations on the part of CHWs for missing incentives for deliveries and to make their income stream less erratic, CHWs should be compensated for pregnancies rather than deliveries. Finally, the program should communicate clearly that the CHW role is voluntary and should develop guidelines in terms of the expected duration of participation for CHWs so that the program and the CHWs have similar expectations.

We explored the impact of dropout of CHWs in two dimensions - firstly, impact of dropout of CHWs on BRAC in terms of cost, from the supply side perspective; and secondly, impact of dropout of CHWs on the community in terms of health services foregone, from the demand side perspective. Dropout is a revealed preference for CHWs themselves since they have better opportunity of earning or they value ‘other’ means of spending their time more, for example, rearing of children, caring for older members of family, and household chores. So, we did not examine this aspect further in this study. We also estimated the cost of the possible strategies to reduce dropout, and compared whether these strategies were more or less costly than the costs borne by BRAC and the community.

## Methods

**Figure 1:**
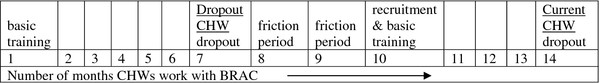
Conceptual timeline of dropout for volunteer CHWs of BRAC, Dhaka urban slums, 2009.

We used a timeline for CHWs working in the community and the events of their recruitment, basic training, refresher training, operations in the community and then their dropout in course of the timeline. At joining each volunteer CHW receives a three-week basic training and attends a one-day refresher training and an incentive day each month. According to the average duration of employment for both the current and the dropout CHWs, potential dropout occurred in the seventh month for the dropout CHWs and in the 14^th^ month for the current CHWs [9]. So, the seventh month and onward for the dropout CHWs, and the 14^th^ month and onward for the current CHWs were the potential friction periods until new recruitments took replace to fill the vacancies of the dropout CHWs (Figure 1).

In order to aid in questionnaire development we conducted explorative field visits and initial interviews with BRAC *Manoshi* Program Manager*,* Senior Health Coordinator, Regional Managers and Branch Managers who executed CHW recruitment, training, development and operations, in order to identify the exact ‘cost inventory’. The overall strategy of costing followed three basic steps: identifying cost inventories; measuring cost items; and valuing them.

### Study sites, respondents and duration

We purposively conducted the study two *Manoshi* field sites of Dhaka urban slums where the program started in January 2007. Based on program data 12 *Manoshi* sites under 12 branch offices were divided into two categories – high performing and low performing sites. Of the two study sites- one represented high performing *Manoshi* field site and another low performing site. There were three sites in which there was complete information and the length of time the program operating was long enough to allow adequate program experience. As of the direction from the advisory committee of *Manoshi* we excluded the third site which was, in fact, high performing but over researched because of the proximity to BRAC Head Quarters. In the two selected study sites we did a complete count rather than adopting any sampling technique. Program Organizers, Branch Managers, Accountants, Regional Managers, Health Coordinator and Program Manager who were directly involved in both financial and program matters regarding recruitment, training, monitoring and supervision of CHWs in the selected study sites were interviewed for collecting CHW related costs and health service information. The reference period for this study was January 2009 to December 2009.

### Estimating the impact of dropout of volunteer CHWs on BRAC

We estimated costs of recruitment, basic training, refresher training and incentive day attended by a CHW using the ‘ingredient approach’ (outlined below). We also estimated average duration of employment of a CHW in the community and average time after basic training until a CHW dropped out. Then we estimated the period of gap in services provided by a CHW in the community after dropout, i.e. how long before a new CHW was recruited and active in the community. Afterwards, we estimated the cost of recruitment and training of a new CHW adapting and applying the so-called ‘friction cost approach’ (outlined below). In addition, we estimated costs of BRAC to operationalize a CHW in the community apart from cost of recruitment and training through reviewing financial documents and interviewing concerned staff members of BRAC.

### Estimating the impact of dropout of volunteer CHWs on the community

We estimated average number of services i.e. number of household visits, pregnancy identification, bringing pregnant mothers to delivery centers, attending deliveries, selling drugs and health commodities, referrals for the complicated cases and other category of health services not provided by a CHW during the ‘friction period’ when a CHW was missing in the community recognizing that these services might have been delivered by other providers of variable quality. We estimated these categories of health services forgone in the community in absolute numbers as an impact of dropout of a CHW. When dropout of a CHW occurs an ad-hoc CHW (a substitute of a dropout CHW) is recruited without basic training. Due to such dropouts, operations are understaffed and the program operates below the standard level. When the program is understaffed, existing CHWs have to share the responsibilities of the missing CHWs. Taking into consideration of the level of staffing and operations capacity we estimated the above missing health services in the community during the friction period using BRAC management information system and monitoring records.

### Estimating cost of possible strategies to reduce dropout of volunteer CHWs

We examined and valued the cost of the strategies identified in an earlier study [11] to reduce dropout applying the ‘ingredient approach’. For example, we estimated the minimum cost of a sari, a pair of shoes and an identity card using the current market price. In this way we estimated the minimum costs for every strategy. Here, the basic assumption was the “minimum cost with maximum retention”. Moreover, all the strategies mentioned were considered together since they were not mutually exclusive.

### The ingredient approach

All relevant resources used in recruitment, basic training, refresher training and average month of work in the community of the CHWs were accounted following the ‘ingredient approach’ [12-15]. The ingredient approach is a standard costing methodology where the researcher examines the process of producing a certain output and lists all the resources or inputs used in the process. The method quantifies all the inputs used in this process irrespective of who provides the input or how the inputs are paid. Here, we considered all possible financial costs based on the cost inventory (salary of staff engaged in recruiting, training, supervising and monitoring of CHWs; rent of *Manoshi* offices used for CHWs, recruitment venue for CHWs, basic training and refresher training venue, transportation, training materials, training and refresher allowance, supply and logistics used for CHWs) related to CHWs. Then we estimated the average cost for a CHW.

### The friction cost approach

We adapted the so-called ‘friction cost approach’ [16] used for measuring the indirect cost of disease (productivity losses) to estimate the cost of dropout of a CHW. The friction cost method does not assume that work is left undone but attempts to calculate the costs of recruiting and training of individuals to keep the work going. Its basic idea is that the amount of production loss due to ‘disease’ (in this study ‘dropout’) depends on the time-span organizations needed to restore the initial production level. In this study, data were collected on when the friction period occurred; how long it lasted; and then the cost during the friction period was estimated. The actual indirect costs of dropout consist of: the value of health services lost and/or the extra cost to maintain health services and, if the CHWs are to be replaced the cost of filling vacancies and training of new CHWs.

### Collecting cost and service information of CHWs

Financial cost and service data were collected by interviewing *Manoshi* staff, extracting from facility records, and observing on the use of space, supplies and furniture used for recruitment, training, development and operations of CHWs in the community using a structured questionnaire. The concerned staff members of the branch, regional and head offices were interviewed to assess the percentage of total time of their involvement with CHWs, and types of activities performed for which there were not likely to be reliable records. The following were the cost categories:

#### Capital costs

Information on the area of branch offices used for CHW related activities were collected. A rough diagram of office floors was drawn on the pages of questionnaire. In the diagram for each of the rooms, the functions normally carried out for CHW related activities were recorded (recruitment venue, training venue, refresher training venue, supervisor’s office and store rooms for training materials and health commodities). Vehicles and different types of furniture, equipment and instruments being used for the CHW activities with their number, life expectancy and market price were collected.

#### Recurrent costs

The questionnaire included all personnel who worked at the *Manoshi* branch office and were involved with CHW related activities during the reference period. Personnel costs were mainly collected based on the information available from the salary register for the reference period, and both program and support staff were asked to find out the percentage of their time they spent during the reference period for CHW related activities. Information on total amount of salary and benefits paid to the staff of branch offices including staff working at regional and head offices for supervising and monitoring of CHWs during the reference period were collected. Information on all types of supplies including drugs, health commodities and training materials were collected. Information on all operations costs including transportation, utilities, maintenance of the branch offices related to CHWs were collected. The equipment, machinery and furniture having a life-span less than one year were not considered as capital items; rather these were considered as recurrent items.

### Allocation of shared costs

Allocation of costs for volunteer CHW related activities were done according to the allocation procedure outlined in Table 1. Personnel cost was allocated based on the percentage of time spent by the program and support staff related to CHWs. Personnel cost was the summation of share of individual’s basic salary and benefits of the reference period. Cost for capital items was allocated to CHW related services, according to their uses and location of services. Cost of office space was apportioned in proportion to area used for CHW related activities. Operations costs including utilities and maintenance of *Manoshi* branch office were apportioned among CHW related activities. Kitchen, training/meeting room and store room were also allocated to CHW related activities. Similar procedures were followed for estimating the costs of recruitment, training and operations of an ad-hoc CHW.

**Table 1:** Allocation of costs for recruitment, training and operations of a volunteer CHW, Dhaka urban slums, 2009

**Cost categories**	**Allocation procedure**
Personnel	Proportion of time spent for CHW related activities
Equipment	Proportion of use of equipment and machineries for CHW related activities
Furniture	Proportion of use of furniture for CHW related activities
Space rent	Proportion of floor space used for CHW related activities
Supplies	Proportion of supplies used for CHW related activities
Operation	Proportion of utilities & maintenance used for CHW related activities
Kitchen	Proportion of use of kitchen for CHW related activities
Transport	Proportion of transport costs incurred for CHW related activities
Training/meeting room	Proportion of training/meeting room costs to CHW related activities
Store room	Proportion of store room used for supplies, training materials and other CHW related activities

### Data analysis, discounting and sensitivity analysis

We analyzed data on all costs and services forgone using Microsoft Excel 2007. Depreciation of capital assets was estimated in order to calculate annual capital costs. For this, we calculated the annualized value using the market price of 2009 as the replacement value. The rate of discount considered for an opportunity cost of the capital inputs was 5% [13]. The average life years for the capital items was considered for the calculations of annualized value. We estimated average cost for a regular CHW and for an ad-hoc CHW. Likewise, we estimated average health services delivered by a regular CHW and by an ad-hoc CHW. We also estimated average cost of possible strategies to reduce dropout of a CHW. We also performed sensitivity analysis of the estimates using the discount rate of 3% [17] and using different rental rates of *Manoshi* office buildings to test the robustness of the cost estimates. Finally, we made descriptive comparisons on whether cost of all strategies reducing dropout of CHWs were more or less than the costs borne by BRAC and the community using all the cost estimates and estimates of health services derived.

The study was approved by the International Centre for Diarrheal Disease Research, Bangladesh (ICDDR,B) Institutional Review Board (Protocol # PR-09078).

## Results

### Impact of dropout of volunteer CHWs on BRAC

Each branch office conducts a needs assessment survey in the respective catchment area before starting formal recruitment process of volunteer CHWs. The survey has two fold objectives – needs assessment of the intervention area and identification of prospective candidates from the community for the positions of volunteer CHWs. After identifying prospective candidates final selection of CHWs is held at the branch office under the active supervision of the respective Branch Manager. In the recruitment process a significant amount of time of *Shasthaya Karmis* (immediate supervisor of CHWs), Program Organizers and Branch Manager is involved. Secondly, office space for interview and the associated recurrent and capital items are involved to furnish recruitment of CHWs. So, the average total cost for personnel, space rent, recurrent and capital items used for the recruitment of a volunteer CHW in 2009 was estimated to be US$ 1.54. On the other hand, when volunteer CHWs drop out ad-hoc CHWs are recruited without any basic training to continue the daily health activities in the community. We estimated that US$ 2.30 was spent on an average to furnish the recruitment process of such an ad-hoc CHW. The reason for higher recruitment cost for an ad-hoc CHW was higher amount of time engagement of the key staff to search and recruit an ad-hoc CHW.

Each volunteer CHW must participate an intensive three-week basic training course which is fundamental for becoming a CHW. CHWs are sent to the basic training in a batch of 20. An ad-hoc CHW also needs to participate in a basic training whenever a batch of 20 volunteer CHWs is formed. This basic training component involves significant investment on the part of BRAC to develop this cadre of community health workforce. The current study estimated the cost involved for the basic training of volunteer CHWs accumulating all the resources used from the supply side. Average cost per basic training of a volunteer CHW was US$ 48.78 after accumulating cost of all human resources, supplies, training venue rent, communication and transportation, and both recurrent and capital items used.

In addition to a basic training, a volunteer CHW needs considerable amount of support and supervision from BRAC staff members while working in the community. If a volunteer CHW works for one month in the community support and supervision from the concerned *Shasthaya Karmi*, Program Organizer, Branch Manager and Regional Manager require at different degree and level. So, the costs of staff-time, their transportation costs and allowances including the apportioned use of capital items like motor bikes were amounted US$ 3.13.

At each month, every volunteer CHW needs to attend a refresher training course and an incentive day at the respective branch office. In both cases, in addition to travel and daily allowances for each CHW a significant amount of staff-time, venue rent, supplies and use of capital and recurrent items occur. Average total cost of all tangible resources for conducting a one-day refresher training for a volunteer CHW was US$ 4.16. Similarly, for participating an incentive day average total cost occurred for a volunteer CHW was US$ 1.67.

So, for all together recruitment, a basic training, a refresher training and an incentive day the amount invested per regular volunteer CHW was US$ 59.28 and for an ad-hoc CHW, the total amount invested was US$ 60.04 (Table 2). The sensitivity analysis revealed that the estimated costs for a regular and an ad-hoc CHW were not sensitive to the changes in the discount rate and *Manoshi* office rental rates used in the analysis.

**Table 2:** Cost of dropout of a volunteer CHW, Dhaka urban slums, 2009

**Cost categories**	**Regular CHW**		**Ad-hoc CHW**	
	**BDT**	**US$**	**BDT**	**US$**
Recruitment of a volunteer CHW	104.63	1.54	156.10	2.30
A three–week basic training	3316.90	48.78	3316.90	48.78
One month work in the community	212.98	3.13	212.98	3.13
One refresher training	283.13	4.16	283.13	4.16
One incentive day	113.63	1.67	113.63	1.67
**Total**	**4031.27**	**59.28**	**4082.74**	**60.04**

*US$ 1 = 68 BDT (Source: www.oanda.com, mid of 2009).

### Impact of dropout of volunteer CHWs on the community

Due to dropout of a volunteer CHW with basic training and subsequent ad-hoc replacement by a CHW without basic training there involves a significant loss of health services on part of the community, the demand side. Due to the absence of a volunteer CHW in the community for her dropout there causes a friction period when the community is deprived of all the community health services received usually from a trained CHW. During this friction period the program initiates replacement of a dropout CHW by an ad-hoc CHW without basic training. According to the program average time needed for such replacement was one month. The community was deprived of the services (Table 3) during this one month or, received services from other sources with variable quality and price. After one month when the replacement took place by a volunteer CHW without basic training, the community was further deprived of the standard services the community used to receive from a trained regular volunteer CHW. An ad-hoc CHW was able to perform equal to 50% of a regular trained CHW in terms of conducting health message session. In selling drugs and health commodities, an ad-hoc CHW was able to sell a little more than 50% of a trained regular CHW did. Similar suboptimal performance also happened in case of distributing iron and folic acid among the pregnant mothers. The reason of such variation was due to lack of basic training and fresh appearance in the community. In case of attending mothers and newborns after delivery, services for neonatal sepsis and birth asphyxia, detecting low birth weight (LBW) and postnatal care services an ad-hoc CHW without basic training performed much lower than that of a trained regular CHW.

**Table 3:** Comparison between a regular and an ad-hoc CHW in providing health services per month, Dhaka urban slums, 2009

**Health services**	**Regular CHW**	**Ad-hoc CHW**
Household visit	200	200
Health message session	40	20
Selling drugs and health commodities (US$*)	7.35	4.41
Pregnancy identification	2	2
Bringing mothers to EPI centre for TT	2	2
Provide iron and folic acid to pregnant mothers	15	8
Bringing mothers to delivery centre	2	2
Attending mothers and newborns after delivery	2	1
Referring complicated cases	1	1
Detect and treat neonatal sepsis and birth asphyxia and refer	<1	0
Bringing 0–1 year babies to EPI centre for immunization	2	2
Ensure vitamin A for undr-5 children in six months	11	11
Detect LBW and educate family members on kangaroo mother care and refer for complications	<1	0.25
Detect and treat ARI and diarrhoea	2	2
Involvement in postnatal care of mothers	2	1

*US$ 1 = 68 BDT (Source: www.oanda.com, mid of 2009).

In addition, the community members are used to be served by regular volunteer CHWs, but their sudden dropout creates anxiety among the community members and it takes time for the community to get adjusted with the substitute ad-hoc CHWs. However, in the current study, we were not able to capture the cost of anxiety of the community.

### Cost of possible strategies to reduce dropout of volunteer CHWs

Out of all possible strategies to reduce dropout, incentive packages from pregnancy identification to attending delivery and newborn care for a single year was US$ 81.18 assuming that a trained regular volunteer CHW with an average performance was able to serve 24 deliveries in a year. In the existing practice a CHW used to receive incentives separately for a) pregnancy identification; b) bringing pregnant mothers to birthing huts; and c) attending the delivery and newborn care. But in the recommended strategy, they would receive the same money at a single time as a single package with the caveat that they would meet certain standards. This would reduce their frustrations of missing incentives if the identified mothers would have delivered in other than BRAC birthing huts.

Increased refresher training allowance would cost US$ 1.18 each month which would be US$ 14.11 in a year. Health insurance could be the most convenient way for ensuring treatment for the family members of volunteer CHWs. Thus providing yearly health insurance premium for a 5-member family with BRAC VO membership would cost US$ 2.21 according to the BRAC micro health insurance project. Cost of two bonuses for a year would be amounted US$ 8.82. Other strategies to reduce dropout of volunteer CHWs would be two saris and two pair of shoes for a year, and an identity card. The average total cost of the recommended strategies was estimated to be US$ 121.28 (Table 4). Last but not the least, clear guidelines for the role of volunteer CHWs and communicating them the guidelines clearly would help the volunteer CHWs to prevent from developing false expectation of being regular staff instead of volunteers.

**Table 4:** Cost of possible strategies to reduce dropout of a volunteer CHW, Dhaka urban slums, 2009

**Strategies to reduce dropout**	**BDT (Yearly)**	**US$ (Yearly)**
Incentive package from pregnancy identification to post-delivery care for 24 pregnancies @BDT 230	5520	81.18
Increased refresher training allowance @BDT 80/month	960	14.11
Health insurance premium for a 5-member VO family @ BDT 150	150	2.21
Bonus before major festivals twice a year @ BDT 300	600	8.82
Two uniform sari @ BDT 300	600	8.82
Two pair of shoes @ BDT 200	400	5.88
One Identity Card @BDT 18	18	0.26
**Total**	**8248**	**121.28**

*US$ 1 = 68 BDT (Source: www.oanda.com, mid of 2009).

## Discussion

Under the current purview of the operations of volunteer CHWs in the community the total investment of BRAC for about 80,0000 volunteer CHWs is huge. In other way, total amount of services provided by BRAC CHWs in the underserved communities in Bangladesh over the years is also enormous in terms of overall health gain and particularly, preventing maternal and neonatal deaths in *Manoshi* project areas. With the current rate of dropout of volunteer CHWs the overall impact of such dropouts is really alarming in terms of absolute loss of financial resources on BRAC side and in terms of forgone health services from the community perspective. In a low income setting, such as Bangladesh where services provided by formal health workers is always limited in addition to inefficient health service management issues. So, the optimum utilization of the invested resources for the informal health workers such as volunteer CHWs is critical to the improvements of the population health in such low income settings.

In case of dropout of volunteer CHWs, BRAC needs to replace them by ad-hoc CHWs who need to be developed through basic training and other subsequent arrangements like refresher trainings and field orientations. So, the investment of BRAC in true financial terms becomes altogether double because of dropout of regular trained CHWs and for keeping the community-based health interventions functional through recruiting and training of ad-hoc CHWs. On the other hand, cost of a set of possible strategies recommended by volunteer CHWs is closely equal to the combined amount of financial loss due to dropout of regular CHWs and subsequent replacement by ad-hoc CHWs and their training. Cost of possible strategies is estimated on yearly basis but 90% of dropouts occur within the period of less than a year and it reflects more costs from dropout if we compare on yearly basis. In addition, there is a good amount of forgone health services in the community because of these dropouts. All these imply high potentials to adopt the recommended strategies and thus reduce dropout of volunteer CHWs. This study explored the scopes of preventing wastage of financial resources with an additional investment in materializing the recommended strategies of reducing dropout. With an additional investment of US$ 121 yearly per CHW BRAC can save another US$ 60 invested for an ad-hoc CHW plus forgone services in the community. It would also ensure the quality of services at standard level which might not be possible in absence of trained volunteer CHWs in the community. So, the findings of the current study has programmatic implications in making the community-based health interventions less erratic but more functional by retaining trained volunteer CHWs.

Issues regarding certain behavioral variables like anxiety of the community members due to sudden dropout of trained CHWs and getting adjusted with the substitute ad-hoc CHWs are difficult to capture without new survey among clients. Mental agony and tension of the community members is important though it has not been possible to cost these mental hazards in true financial terms. Such intangible costs arising from mental hazards are seldom reported in literature because of its difficulty to measure [18,19]. Though the anxiety in the community or program is not quantified in this current study, retention of CHWs certainly puts additional benefit reducing anxiety. The overall gain in preventing dropout of volunteer CHWs through adopting recommended strategies is absolutely higher than the loss due to dropout.

This study has important implications for making both BRAC volunteer CHW model and community-based health interventions sustainable. Since BRAC is working in several parts of Asia and Africa in low income settings using the similar volunteer CHW model, the findings of the current study has significant implications in ensuring better utilization of the invested resources in developing and training of such health cadres. Adopting the simple strategies retention of such health workforce, which is often low, could be immensely improved. This improved retention of the grass root health care providers would accelerate the positive health outcomes and would contribute to reducing the disease burden at population level. At the same time, positive health gain by preventing forgone health services in the communities is possible at the cost of these very simple strategies. Up-keeping trust of the community through such low-cost health services, which is often in jeopardy due to frequent dropout of community health workforce, is also important for the success of community-based health interventions. Preventing dropout of CHWs could reduce tension of the community resulting from their dropout and simultaneously, could maintain the quality of health services in the community. So, significant reduction in dropout of CHWs and subsequent improvement in their retention could be an important factor of success of community-based health interventions.

Exercises around the current study also demonstrate the feasibility of applying the so-called ‘friction cost approach’ in measuring the impact of dropout of workforce in other non-health settings. This aspect of the current study is important contribution to new knowledge through applying methodology of indirect cost estimation of diseases in health workforce research, in a non-disease setting. Although the use of the above methodologies particularly, the so-called ‘friction cost approach’ on practical ground in a non-health setting was challenging, it demonstrated new ways and provided new insights in applying critical methodologies in health service research.

## Conclusion

Although CHWs work as volunteers in Dhaka urban slums impact of their dropout is high both in financial term and forgone services. Cost of dropout of female volunteer CHWs of BRAC is almost equal to the cost for retaining them adopting the recommended strategies. Additionally, BRAC can save the foregone health services in the community at the cost of adopting the strategies of retention for these CHWs. High costs of dropout of CHWs along without implementing of strategies of reducing dropout make the program less sustainable. However, there are simple and financially competitive ways which BRAC can consider to improve the sustainability of the volunteer CHW model as well as the community-based health program where they work.

Retention of lay community health workforce is crucial to achieving the universal health coverage in a low income setting like Bangladesh. In order to improve retention of the existing female volunteer CHWs of BRAC, the current study is an important step to inform the BRAC Health Program of the impact of CHWs’ dropout. Information and knowledge on the impact of dropout of CHWs would guide the health program managers and policy makers increasing their awareness and sensitivity on this very issue of dropout of CHWs and thereby, would instigate programmatic actions to reduce such dropouts. Such programmatic actions would contribute to achieving universal health coverage and subsequently gaining improved health outcomes in Bangladesh and other developing countries where volunteer CHWs are commonly used to serve the under privileged communities.

## Competing interests

None declared. Both KA and JAMK are working for ICDDR,B but recently KA is on leave from ICDDR,B for his PhD study at Monash School of Public Health & Preventive Medicine, Monash University, Melbourne, Australia. While designing this study DGW was the Associate Professor of International Health Department at Johns Hopkins University, USA and later he joined Bill & Melinda Gates Foundation. DGW worked on *Manoshi* project as a part-time consultant.

## Authors’ contributions

KA conceptualized the research project in consultations with DGW. JAMK was involved in designing and implementing the research protocol. KA collected data, analyzed and prepared the draft. JAMK and DGW provided feedbacks to shape the final manuscript. All authors read and approved the final manuscript.

## Pre-publication history

The pre-publication history for this paper can be accessed here:

http://www.biomedcentral.com/1472-6963/12/260/prepub
